# Dual hydration of oceanic lithosphere

**DOI:** 10.1093/nsr/nwad251

**Published:** 2023-09-22

**Authors:** Fan Zhang, Jian Lin, Rixiang Zhu, Xubo Zhang, Jiangyang Zhang, Zhiyuan Zhou

**Affiliations:** Key Laboratory of Ocean and Marginal Sea Geology, South China Sea Institute of Oceanology, Chinese Academy of Sciences, China; Department of Ocean Science and Engineering, Southern University of Science and Technology, China; Key Laboratory of Ocean and Marginal Sea Geology, South China Sea Institute of Oceanology, Chinese Academy of Sciences, China; China-Pakistan Joint Research Center on Earth Sciences, CAS-HEC, Pakistan; Institute of Geology and Geophysics, Chinese Academy of Sciences, China; Key Laboratory of Ocean and Marginal Sea Geology, South China Sea Institute of Oceanology, Chinese Academy of Sciences, China; China-Pakistan Joint Research Center on Earth Sciences, CAS-HEC, Pakistan; Key Laboratory of Ocean and Marginal Sea Geology, South China Sea Institute of Oceanology, Chinese Academy of Sciences, China; China-Pakistan Joint Research Center on Earth Sciences, CAS-HEC, Pakistan; Department of Ocean Science and Engineering, Southern University of Science and Technology, China

## Abstract

Water input budget of global oceanic lithosphere at different tectonic settings are quantitatively estimated. The results indicate that the hydration at subduction zone is fundamentally essential to plate dynamics and water cycle of the Earth.

Water is crucial to processes of both Earth's surface and interior, facilitating plate tectonics and making the Earth a habitable planet [1]. Seawater penetrates into oceanic lithosphere through fractures on seafloor and migrates along faults and pores. The hydration process weakens the crustal strength and alters mantle properties. Specifically, it influences seismicity by changing the pressure and friction of rocks, and triggers magma generation by lowering the mantle solidus and mantle viscosity. The distribution of water governs the style of convective flow and distinct tectonic behavior of the Earth [2]. Hence, a quantitative estimation of the water input budget of oceanic lithosphere is required to study the water cycle between the surface and the deep mantle.

The hydration extent of oceanic lithosphere has been estimated by using various methods, including magnetotelluric/controlled source electromagnetic experiments, passive/active seismic expeditions, thermopetrological modellings and laboratory measurements of mineral geophysical and geochemical properties. The results have shown the hydration degree of the oceanic crust is varying during its life cycle. However, the geographical distribution and inventory of the water input budget of the oceanic lithosphere at different tectonic settings remain unclear, especially for the mid-ocean ridge (MOR) and oceanic transform fault (OTF) systems.

The hydration of the oceanic lithosphere is composed of two key stages (Fig. 1a). The ‘initial’ hydration mainly occurs at MORs, where water percolates into newly formed oceanic crust. Additional hydration occurs in upper mantle at OTFs. When the oceanic plate moves away from the ridge axis, the subsequently accumulated sediments blanket the seafloor and keep the water in oceanic crust. The ‘secondary hydration’ occurs at subduction zones, where water percolates into crust and upper mantle through extensional faults in response to plate bending. Seawater reacts with minerals within the plate and causes extensive serpentinization, which lowers the lithospheric strength and facilitates extensional earthquakes, thus further enhancing hydration in the deep portion of the subducting plate. Hydration also occurs at fracture zones and seamounts, which provide extra channels for water infiltration. The water input rates for different tectonic settings are controlled by the length or width of the plate boundaries, the properties of hydrated lithologic layers (e.g. bulk H_2_O content, density and thickness), the spreading rate at MORs, as well as the convergence rate and plate age at subduction zones.

**Figure 1.**
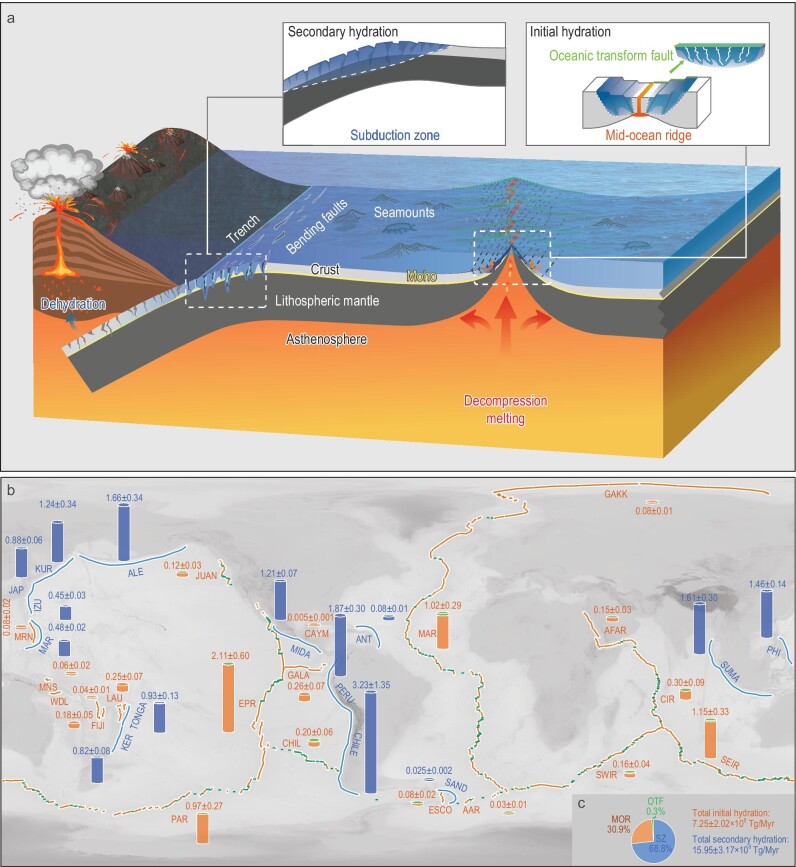
(a) Schematic map of hydration of oceanic lithosphere at mid-ocean ridges (MORs), oceanic transform faults (OTFs) and subduction zones (SZs). (b) Global distributions of water input rates at MORs, OTFs and SZs. MORs and OTFs are marked as orange and green curves: JUAN (Juan de Fuca Ridge), EPR (East Pacific Rise), GALA (Galapagos Ridge), CHIL (Chile Ridge), PAR (Pacific-Antarctic Ridge), MRN (Mariana Ridge), MNS (Manus Ridge), WDL (Woodlark Ridge), FIJI (Fiji Ridge) and LAU (Lau Ridge), GAKK (Gakkel Ridge), MAR (Mid-Atlantic Ridge), AAR (American-Antarctic Ridge), CAYM (Cayman Ridge), ESCO (East Scotia Ridge), AFAR (African-Arabian Ridge), CIR (Central Indian Ridge), SEIR (Southeast Indian Ridge) and SWIR (Southwest Indian Ridge). SZs are marked as blue curves: MAR (Mariana), IZU (Izu-Bonin), JAP (Japan), KUR (Kuril), Aleutian (ALE), MIDA (Mid-American), PERU (Peru), CHILE (Chile), TONGA (Tonga), KER (Kermadec), PHI (Philippine), ANT (Antilles), SUMA (Sumatra) and SAND (South Sandwich). (c) The relative proportion of water input rates of MORs, OTFs and SZs, respectively.

At MORs, seawater normally penetrates the oceanic crust and causes hydration of the oceanic crust mainly within ages of 10 Myr through fractures at both flanks of the ridge axis [3]. The water input rate of each lithologic layer for a MOR segment is calculated by using


(1) \begin{eqnarray*}
Q_{{H}_2O}^{MOR} &=& C_{{H}_2O}^{bulk} \times {\rho }_{layer} \times {H}_{layer}\\
&& \times \, \sum\limits_{i = 1}^N {{v}_{spr}^{i}{l}^i},
\end{eqnarray*}

where $C_{{H}_2O}^{bulk}$ is the bulk H_2_O content in the lithologic layer (wt%), ${\rho }_{layer}$ is its density (kg/m^3^), ${H}_{layer}$ is its thickness (km), ${v}_{spr}$ is the full spreading rate (mm/yr), *i* is the number of MOR segments and *l* is the length of each MOR segment [4]. The water input of the sediment layer is not included in this study. For the basalt and gabbro layers of the crust, the bulk H_2_O contents are assumed as 2.5 and 0.8 wt%, respectively [5], and the bulk H_2_O content of the serpentinized mantle layer is 6.0 wt% [6]. A small fraction of ridge segment could suffer strong seafloor hydrothermal alteration. The bulk H_2_O content of the upper basalt crust for these ridge segments of very limited length increased by one or two times and this may slightly affect the water input rate of global MOR systems [7,8]. The corresponding densities of basalt, gabbro and serpentinized mantle layers are set as 2800, 3000 and 2870 kg/m^3^, respectively. At ultra-slow spreading ridges with *v_spr_* of <20 mm/yr, mantle is directly exposed to the seafloor at some amagmatic segments and serpentinized, but the proportion of segments with serpentinized mantle is not well constrained. For simplicity, we assume that mantle is directly exposed to the seafloor at all ultra-slow spreading ridge segments with *v_spr_* of <10 mm/yr and the average thickness of the serpentinized mantle is 4 km [9]. For other spreading ridges with *v_spr_* of >10 mm/yr, it is assumed that no mantle is serpentinized and the average thicknesses of basalt and gabbro layers of the crust are assumed as 1.84 and 4.31 km [3], respectively.

In addition, the OTFs provide extra pathways for seawater to penetrate into the upper mantle. The mantle serpentinization depth is constrained by the 800°C isotherm that is predicted by the half space cooling model. The water input rate of the overlying crust has been calculated for the MOR scenario and the extra contribution of serpentinized mantle layer for the OTF is calculated by using


(2) \begin{eqnarray*}
{Q}\, _{{H}_2O}^{OTF} &=& \frac{1}{2}C_{{H}_2O}^{bulk} \times {\rho }_{layer} \times {H}_{layer}\\
&& \times \sum\limits_{i = 1}^N {\frac{{{v}_{spr}^{i}}}{2}} {W}_{OTF}^{i},
\end{eqnarray*}

where ${H}_{layer}$ is the thickness of the serpentinized mantle layer, which is calculated by subtracting the average crustal thickness (6.15 km) from the estimated mantle serpentinization depth; *W_OTF_* is the width of the OTF [10,11]; and $\frac{{{V}_{spr}}}{2}$ represents the half spreading rate. The OTF cross section was simplified as a triangle, which introduces a $\frac{1}{2}$ into the area calculation of the cross section. As the underlying mantle is covered by faulted oceanic crust rather than directly exposed to the seafloor, the bulk H_2_O content of the serpentinized mantle layer is assumed as 2.0 wt% and the corresponding density is 3140 kg/m^3^ for the tectonic settings of OTFs and subduction zones [5]. The bulk H_2_O contents and densities of crustal layers for the OTFs and subduction zones are the same as those of MORs.

For global MORs and OTFs, the total water input rate is ∼7.25 ± 2.02 × 10^8^ Tg/Myr (Fig. 1b and Supplementary Tables S1, S2 and S4), which accounts for ∼31.2% of the global water input rate (Fig. 1c). Within the width range of the OTF, a part of the water input rate (∼0.29 × 10^8^ Tg/Myr, 1.3% of global water input rate) is inherited from the crust layer of MORs. Solely for OTFs, the water input rate of the mantle layer is ∼0.07 × 10^8^ Tg/Myr (0.3% of the global water input rate). The Pacific, Indian and Atlantic & Arctic Oceans contributed 59.2%, 24.2% and 16.6% of the water input rate of MORs, respectively (Supplementary Fig. S1a). The Pacific, Indian and Atlantic & Arctic Oceans contributed 28.6%, 28.6% and 42.8% of the water input rate contributed by serpentinized mantle of OTFs, respectively (Supplementary Fig. S1b).

Oceanic lithosphere is further hydrated at subduction zones. The subducting plates bend and generate pervasive normal faults that cut through the oceanic crust and upper mantle, providing pathways for seawater infiltration in the outer rise area. As the oceanic crust has been hydrated at MORs, we assume that the hydration state of subducting oceanic crust is inherited from MORs and the additional hydration only occurs in the mantle layer. The extent of hydration of the subducting plate is related to the age of the plate. For the older subducting plates, bending-related normal faults could cut into deep depth and induce a great degree of hydration. In contrast, the hydration degree of the younger subducting plates is relatively low and limited to shallow depth.

The possible maximum hydration zone of the subducting plate is constrained by the brittle yield zone (BYZ), which defines the possible area where faults could develop (Fig. 1a). The depth of the BYZ can be estimated by comparing the bending stress and the yield strength envelope of the oceanic lithosphere [12]. It is also constrained by the depth at which seismic velocity decreases. Considering differences in the length of subduction zones and the subduction rates, the water input rate of each lithologic layer for a subduction zone is computed as


(3) \begin{eqnarray*}
{Q}\, _{{H}_2O}^{SZ} &=& C_{{H}_2O}^{bulk} \times {\rho }_{layer} \times {H}_{layer} \\
&&\times \sum\limits_{i = 1}^N {{v}_{sub}^{i} {l}^i} ,
\end{eqnarray*}

where $C_{{H}_2O}^{bulk}$ is the bulk H_2_O content in the lithologic layer (wt%), ${\rho }_{layer}$ is its density (kg/m^3^), ${H}_{layer}$ is its thickness (km), ${v}_{sub}$ is the subduction rate (mm/yr) of a subduction zone segment and *l* is the length of a subduction zone segment. The thickness of serpentinized mantle is determined by subtracting the reference crustal thickness (6.15 km) from the average depth of the BYZ.

The additional water input rate contributed by serpentinized mantle of global subduction zones is ∼15.95 ± 3.17 × 10^8^ Tg/Myr (Fig. 1b and Supplementary Tables S3 and S4), which accounts for ∼68.8% of the global water input rate (Fig. 1c). The Pacific, Indian and Atlantic & Arctic Oceans contributed 89.2%, 10.1% and 0.7% of the water input rate contributed by serpentinized mantle of subduction zones, respectively (Supplementary Fig. S1c). The result is consistent with the water input rate estimation obtained from thermopetrological models [5].

In summary, for the crustal layer of oceanic lithosphere, the water input rate of global MORs is ∼7.08 × 10^8^ Tg/Myr. For the mantle layer of oceanic lithosphere, the water input rate of global MORs and OTFs is ∼0.17 × 10^8^ Tg/Myr, and the water input rate of subduction zones is ∼15.95 × 10^8^ Tg/Myr, respectively. The results provide a first-order approximation. However, only a few sections have been sampled or imaged, and any general assumptions for the thickness and bulk H_2_O content of each lithologic layer are oversimplified, thus uncertainties are inevitable.

The results indicate that ∼98.9% of mantle hydration is contributed by the subduction-related process. As a plate subducts into the mantle, some of the water hosted in the subducting plate will be degassed and the remaining is transported to various depths of Earth's interior. The water stored in the deep will also return to the surface through intraplate volcanisms and magmatisms at MORs and OTFs.

The critical role of water in deep mantle has been intensively investigated and summarized, indicating that water could influence the plate tectonics and mantle convection, facilitate subduction initiation and play an important role in earthquakes and volcanism [1]. In this study, we evaluate the dual hydration of oceanic lithosphere and emphasize that the secondary hydration at the subduction zone is fundamentally essential to the plate dynamics and water cycle of Earth.

## Supplementary Material

nwad251_Supplemental_Files
